# Gene Presence/Absence Variation analysis of coronavirus family displays its pan-genomic diversity

**DOI:** 10.7150/ijbs.58220

**Published:** 2021-08-27

**Authors:** Du Jiao, Xiaorui Dong, Yingyan Yu, Chaochun Wei

**Affiliations:** 1 Department of Bioinformatics and Biostatistics, School of Life Sciences and Biotechnology, Shanghai Jiao Tong University, 800 Dongchuan Road, Shanghai 200240, China.; 2 Department of General Surgery of Ruijin Hospital, Shanghai Institute of Digestive Surgery, and Shanghai Key Laboratory for Gastric Neoplasms, Shanghai Jiao Tong University School of Medicine, 200025, Shanghai, China.; 3 SJTU-Yale Joint Center for Biostatistics and Data Science, Shanghai Jiao Tong University, 800 Dongchuan Road, Shanghai 200240, China.

**Keywords:** COVID-19, SARS-CoV-2, Genome, Diversity, Pangenomics

## Abstract

SARS-CoV-2 belongs to the coronavirus family. Comparing genomic features of viral genomes of coronavirus family can improve our understanding about SARS-CoV-2. Here we present the first pan-genome analysis of 3,932 whole genomes of 101 species out of 4 genera from the coronavirus family. We found that a total of 181 genes in the pan-genome of coronavirus family, among which only 3 genes, the S gene, M gene and N gene, are highly conserved. We also constructed a pan-genome from 23,539 whole genomes of SARS-CoV-2. There are 13 genes in total in the SARS-CoV-2 pan-genome. All of the 13 genes are core genes for SARS-CoV-2. The pan-genome of coronaviruses shows a lower level of diversity than the pan-genomes of other RNA viruses, which contain no core gene. The three highly conserved genes in coronavirus family, which are also core genes in SARS-CoV-2 pan-genome, could be potential targets in developing nucleic acid diagnostic reagents with a decreased possibility of cross-reaction with other coronavirus species.

## Introduction

Coronaviruses can cause respiratory and intestinal infections in animals and humans. They are the largest RNA viruses which include four genera (α-, β-, γ-, and δ-coronaviruses) in their family 1. The α-coronavirus and β-coronavirus are recognized as pathogens of mammals, especially causing human respiratory diseases and gastroenteritis, while the γ-coronavirus and δ-coronavirus are considered as pathogens of birds, but a few of them are pathogenic to mammals as well^1^, ^2^. Some of the β-coronaviruses contribute a tremendous threat to the human community. In 2002, severe acute respiratory syndrome (SARS) caused by coronavirus (SARS-CoV) broke out in Guangdong Province, China. Ten years later, another highly pathogenic coronavirus, the Middle East Respiratory Syndrome Coronavirus (MERS-CoV), appeared in Middle Eastern countries 3. Many studies have found that SARS-CoV is transmitted from civet cats to humans, while MERS-CoV is transmitted from dromedaries to humans 1, 4-6. In December 2019, a large-scale respiratory disease caused by coronavirus broke out in Wuhan, China. The pathogenic virus is a coronavirus, but different from the previous two coronaviruses. The International Committee on Taxonomy of Viruses (ICTV) named this new coronavirus as "SARS-CoV-2" 7. Hitherto, there are seven types of coronaviruses that can infect humans. The three viruses mentioned above mainly cause respiratory infections in humans, and other four types of coronaviruses including HCoV-229E, HCoV-OC43, HCoV-NL63 and HCoV-HKU1 can cause mild respiratory diseases, or serious infections in immunocompromised peoples such as infants and elderlies 8, 9. In nature, there are many other types of coronaviruses that can infect poultries or mammals 10.

The concept of pangenomics was proposed by Tettelin et al. 15 years ago 11. Pangenomics describes the union of sequence entities (usually genes or open reading frames, ORFs) shared by genomes of interest 12. If a gene exists in all individuals of a population (such as a specific species), it is called as a core gene. The core genes are usually responsible for the main phenotypes of the species, and are mostly the housekeeping genes. If a gene does not exist in all individuals of a specific species, it is called a distributed gene or a dispensable gene, which is considered as non-essential for life activities, but contributes to species diversity and adaptation to the environments with some selective advantages 13. Recently, Wang and colleagues reported pangenomic features of African swine fever virus genomes, and proposed that the pan-genome of African swine fever viruses was "open", indicating an extremely large number of genes. This finding will provide important reference for vaccine development 14. With the continuous growth of whole genome sequencing data, pangenomics analysis has been widely used in the field of microbiological genome researches.

Since the outbreak of COVID-19 at the end of 2019, a large number of studies on coronavirus have been published 15-20. Thanks to the continuous uploading of the genome-wide sequencing data of the virus to public databases, the genome-wide sequencing data of coronaviruses are exploding. These data provide a good opportunity for pangenomics analysis of coronavirus family. Since the pan-genome could provide a genomic overview of the whole coronavirus family, including the core genes and dispensable genes, it should help researchers to clarify the phylogenic relationships and genetic diversity of the coronaviruses. These problems have important clinical implications, and are related to pathogenicity, immune escaping, vaccine design, nucleic acid detection, and drug development 21. Therefore, we did pangenomic analysis of coronavirus family in this study. A total of 4,142 viral genomes covering four genera from coronavirus family were downloaded from public databases.

In order to validate the analytic results of pangenomics study, ortholog analysis was introduced. Orthologous analysis is similar to sequence similarity analysis, which can be used to reconstruct the evolutionary relationship of microbial genomes 22, and orthologous analysis can also infer the common ancestor between different species 23. Therefore, the combination of pangenomic analysis and ortholog analysis will improve the reliability. In this study, we used this combined strategy.

## Results

### Pangenomic characterization of the coronavirus family

We constructed the pan-genome of coronaviruses family from 3,932 coronavirus genome sequences (Table S1). At the identity percentage threshold of 30%, 181 different genes were observed in coronavirus family** (**Table S2). Under this threshold, we found three highly conserved genes that were present in more than 97% of the individual genomes. They are spike protein encoding gene (S), membrane protein encoding gene (M), and nucleocapsid protein encoding gene (N). The NCBI annotation information and Roary annotation information of genes (see Methods for details) that existed in more than 1,000 coronaviruses genomes were listed in Table 1. The S gene was present in almost over 99% of coronavirus genomes, while the M gene and N gene presented in over 97% of coronavirus genomes, indicating that they were highly conserved genes in the coronavirus' family. The presence and absence variation (PAV) of coronavirus family is shown in Figure 2.

**Figure 1:**
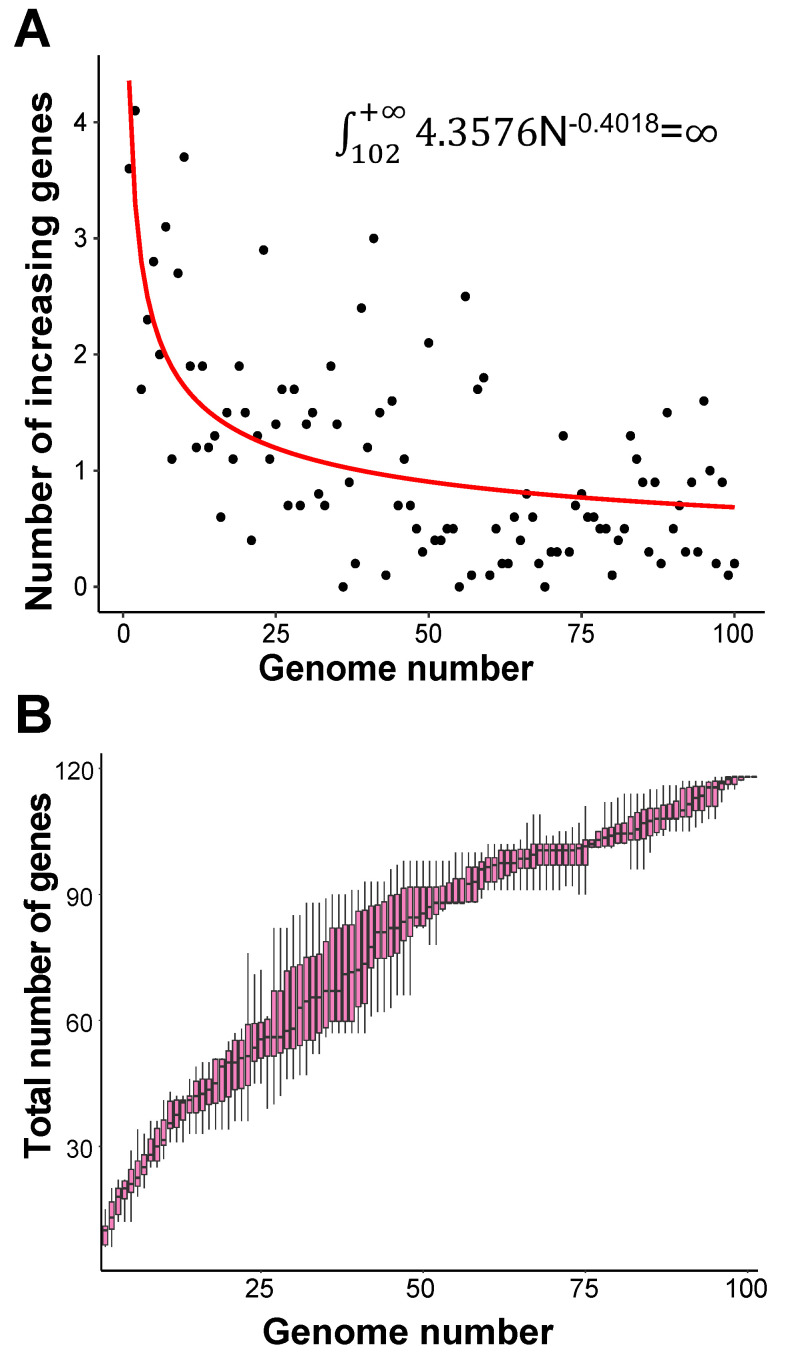
**Pan-genome size estimation for the coronavirus family.** A. Estimating the pan-genome size of the coronavirus family based on a power function (n=kN^r^), where n represents the number of additional new genes detected for each additional viral genome sample, N represents the number of genome samples, and k and r are the two parameters of the model. The points used for fitting are the average values of 10 random sampling of one additional genome. The fitting result is k = 4.358 (3.501, 5.263); r = -0.402 (-0.471, -0.330) (adjusted R^2^ = 0.45). The values in brackets are 95% confidence intervals. B. The trend of total number of genes of the coronavirus family when the number of genomes increases. Along with the increasing number of genomes, the pan-genome size continues to increase, and does not reach a plateau stage.

In order to evaluate the “open” or “closed” status of the pan-genome of coronavirus' family, we sampled various numbers (from 1 to 101, with an increment of 1 each time) of genomes from the “101 data set” (See Data collection section of Methods for more details), and for each number of genomes, we randomly resampled 10 times. Power regression (n = κN^α^) was conducted to determine the number of new genes detected for each additional genome, using the average number of extra genes derived from a new genome newly added. The fitting curve function is n = 4.3576N^-0.4018^ (parameter t test p<0.0001, adjR^2^=0.45), as shown in Figure 1A. Therefore, the total number of undiscovered genes in the 101 species is 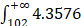
N^-0.4018^=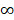, suggesting that the pan-genome of the coronavirus family is “open” (Figure 1).

**Figure 2:**
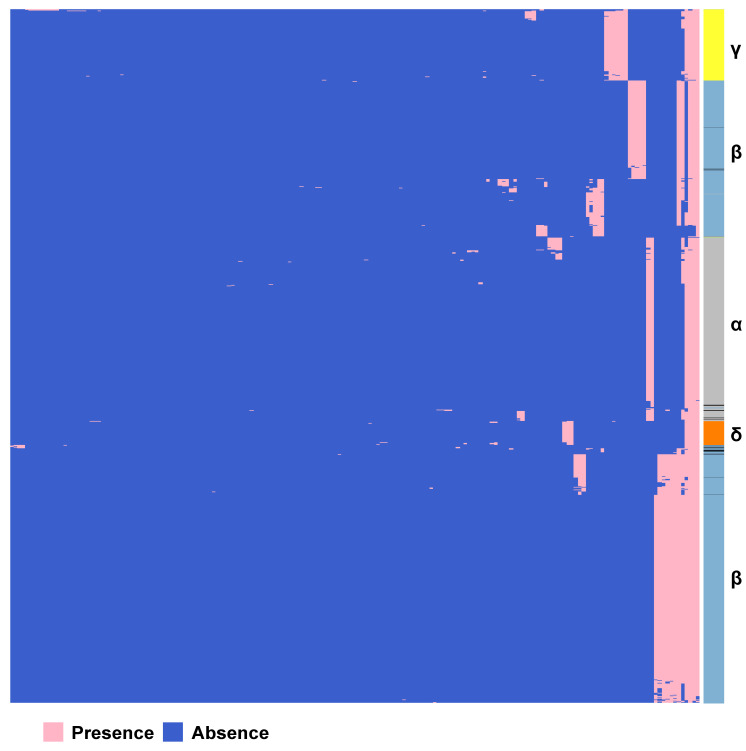
** Gene PAVs from pan-genomics analysis of 3,932 genomes of the coronavirus family.** The right side coloured partitions represent different genera, and each row is for a genome. Black represents unclassified genera. The horizontal axis stands for the 181 genes in coronavirus family derived from 3,932 genome sequences when the sequence identity percentage was set to 30%.

**Figure 3:**
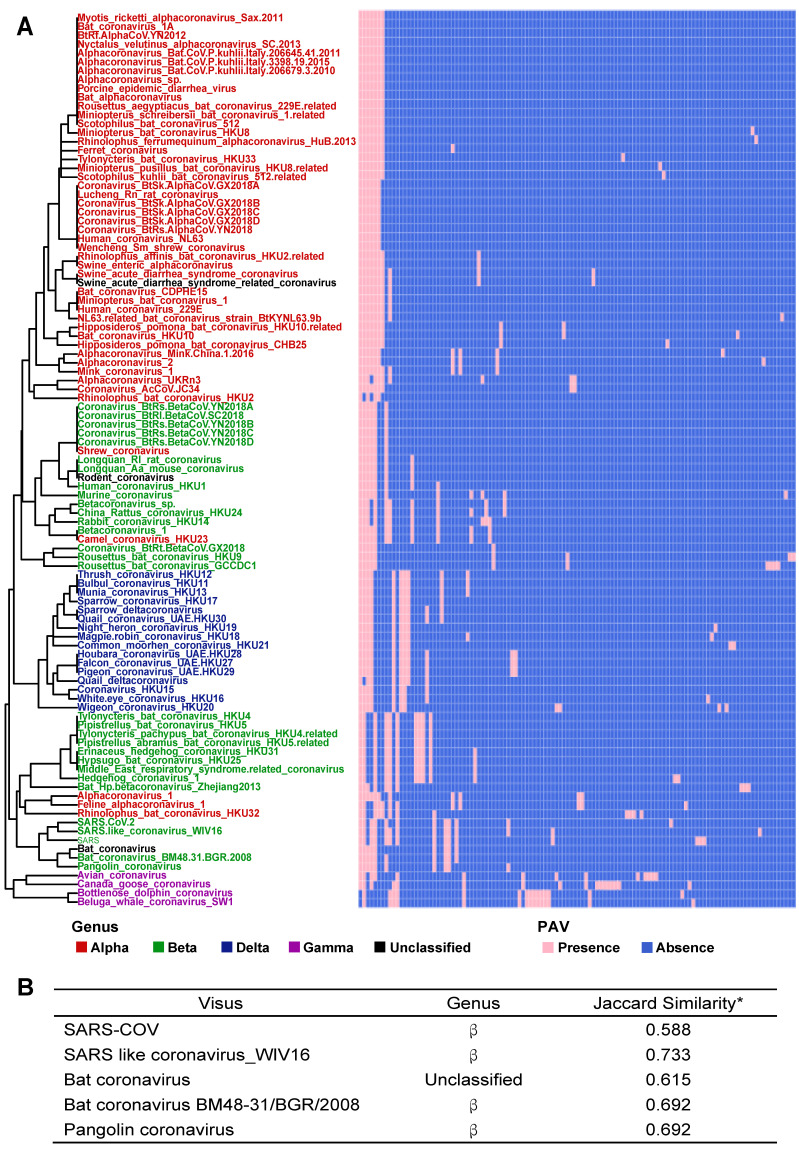
**The PAVs of 101 species of coronavirus family and the interspecies similarity analysis to SARS-CoV-2 sequence**. A. Each row represents a species, and each column represents a gene. There are 101 species and 181 genes in total. Pink represents the presence of genes, and blue represents the absence of genes. In the left clustered tree, different genera are represented by different colors. B. Viral genomes similar to SARS-CoV-2 coronavirus genome. *The similarity value is in [0, 1], and the larger the value, the higher the similarity. Only the names of species with similarity greater than 0.5 are listed.

According to PAV analysis, the presence and absence patterns of coronavirus genes in different genera are quite different. However, there are commonalities within the same genus. The genes inside α genus are basically the same, and so are γ and δ. The genes within β genus are somewhat different (Figure 2). There are few highly conserved genes in the genomes of the coronavirus' family, and most genes are unique to an individual or a specific species. Each genus has its own unique genes. For example, ORF8 and ORF10 genes are specific genes of β-coronavirus. We noticed that Bat_coronavirus (unclassified virus in NCBI) was close to β-coronavirus, and genes ORF3a, ORF6 and ORF7b are also specific genes for β-coronavirus (Table 1). We further clustered the genomes from the “101 data set” (See Methods for detailed definition), and clearly found that genes in SARS genomes, SARS-like virus genomes, and coronavirus genomes from bats are relatively close to SARS-CoV-2 genome (Figure 3).

Based on the Jaccard similarity (see Methods for details), we calculated the similarity between 101 species of the coronavirus family (Table S3). We found that the similarity coefficients between many species are very low (<0.25), indicating that there is a certain degree of diversity among the genomes of different species. We further calculated the similarity coefficients between the genomes of the same species and compared them with the similarity coefficients between the genomes of different species. Statistical tests showed significant differences (Wilcoxon test, p<0.0001). Therefore, we believe that there are significant differences between the genomes of different species.

### Pangenomics characterization of SARS-CoV-2 genomes

We further analysed the pangenomic features of 23,539 SARS-CoV-2 genomes with high quality (Table S4). There are 13 genes in SARS-CoV-2 pan-genome. The 23,539 SARS-CoV-2 genomes were sampled from different countries and regions. We outlined 13 core genes for SARS-CoV-2 genome, which means the conservation level of SARS-CoV-2 genomes is high.

### Orthologous analysis of proteins encoded by coronavirus genes

**Figure 5:**
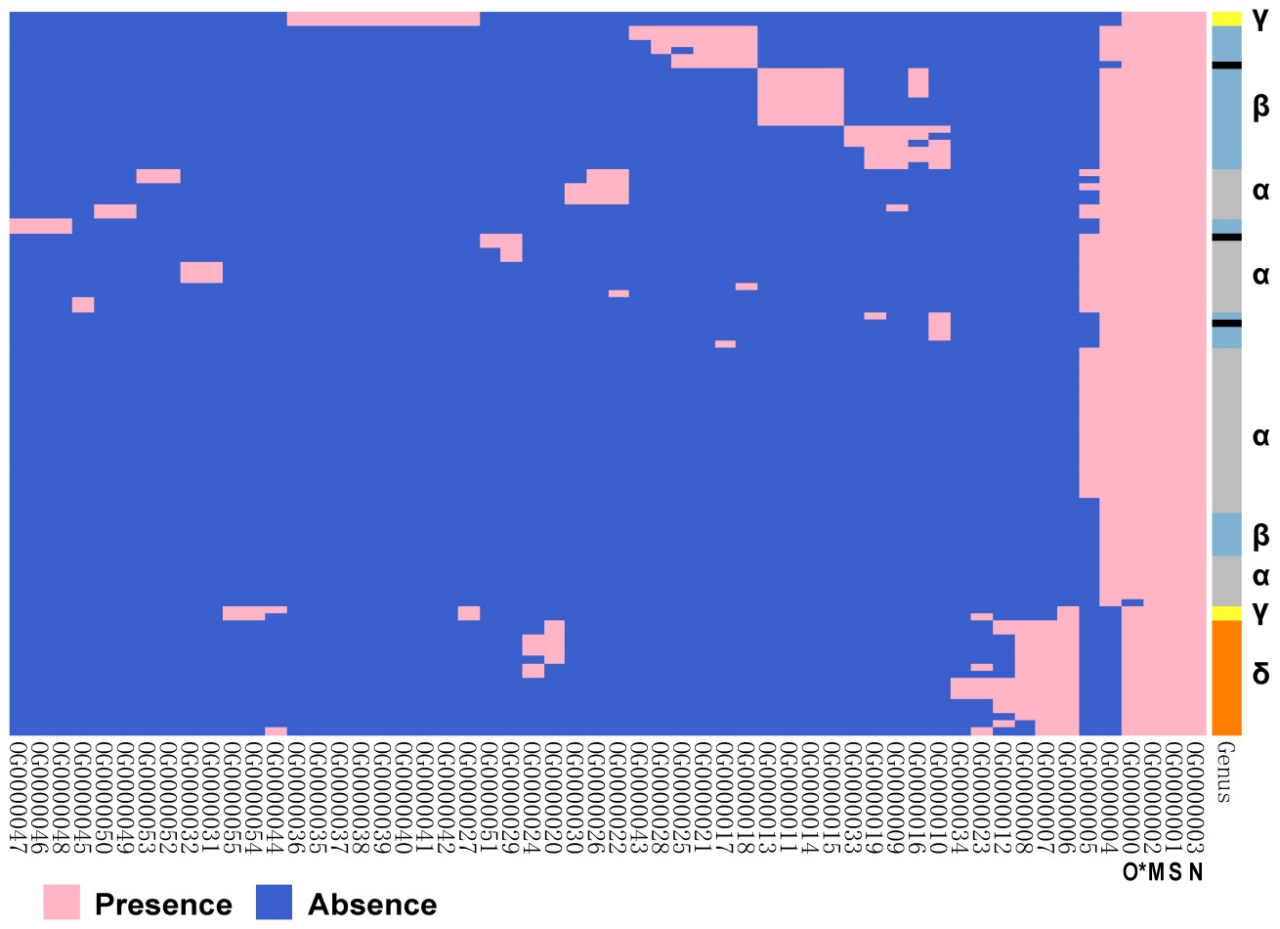
**The ortholog analysis of proteins encoded by coronavirus genes.** The right coloured partitions represent different genera, and each row is for a genome. Black represents unclassified genera. Each column represents an orthologous protein, each row represents a virus species, pink represents the presence of protein, and blue represents the absence of protein. The naming of orthologous proteins is defined by Orthofinder. (* O stands for ORF1ab protein.)

The orthologous proteins of 101 species of coronavirus genomes were analysed (Figure 5). There are three conserved proteins in each virus genome. They are proteins OG0000001~OG0000003, which correspond to proteins encoded by S gene, M gene, and N gene. The protein OG0000000 corresponds to protein encoded by ORF1ab gene, which is found in over 99% of viral genomes. The results implied that these proteins may come from a common ancestor, which suggests that the whole family of coronaviruses originated from a same viral species. On the other hand, we noticed that protein OG0000004 was encoded by gene E, which was obviously absent in γ genus and δ genus.

**Figure 6:**
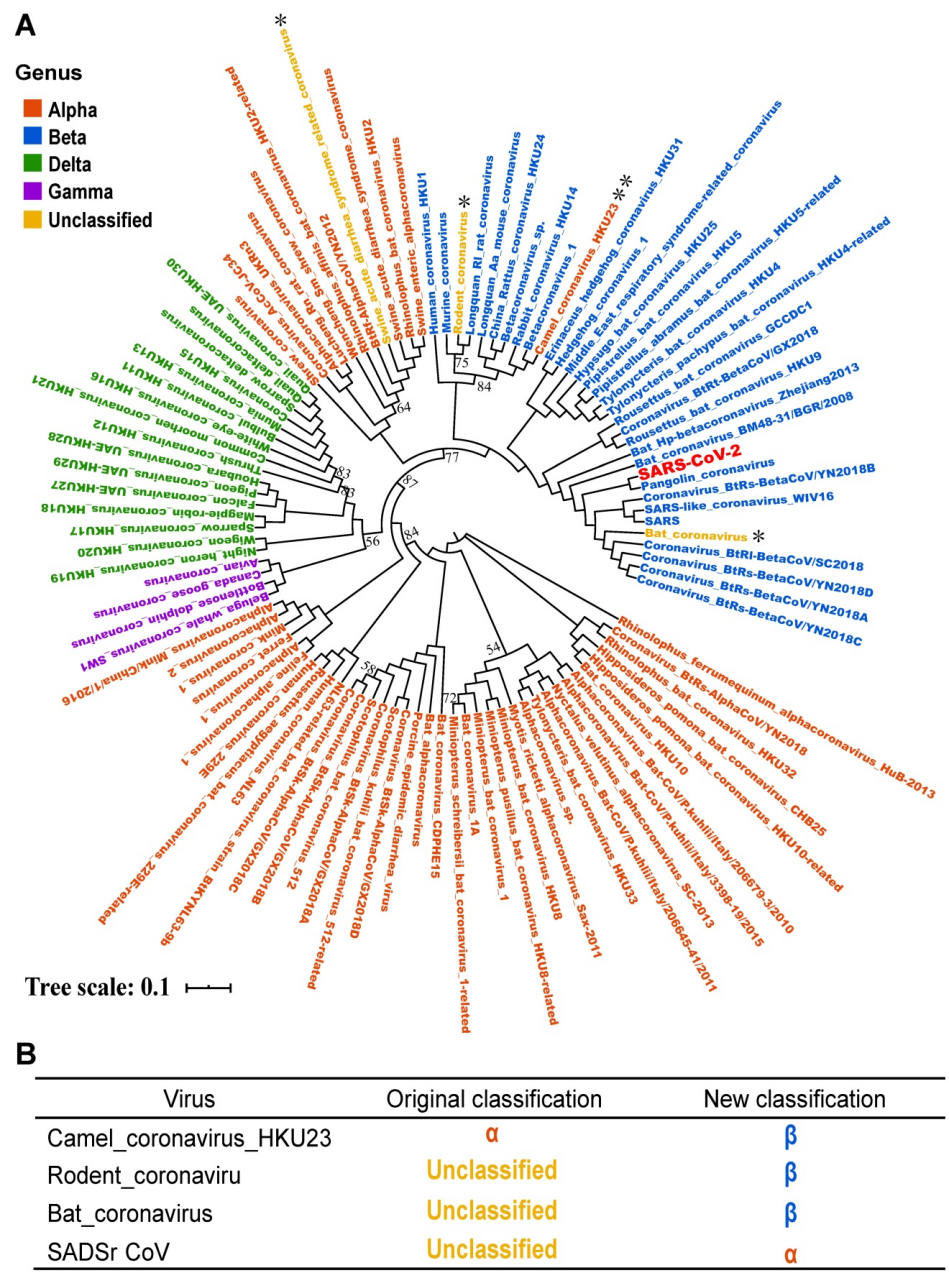
** Phylogenetic tree analysis of the coronavirus family.** A. Using iqtree's maximum likelihood method to infer the phylogenetic tree, and bootstrap is set to 1000. Different colors in the phylogenetic tree represent different genera. The branches with bootstrap values less than 90 are marked with bootstrap values, and the bootstrap values for the remaining branches are all 100. B. The four coronaviruses reclassified through our research. The colors in the second and third columns of the table are consistent with colours of genera in the phylogenetic tree.

Based on above orthologous protein analysis, we constructed the phylogenetic tree of coronaviruses family (Figure 6). SARS, Bat coronavirus BM48.31.BGR.2008, Coronavirus_BtRs-BetaCoV/YN2018B and Bat HP-betacoronavirus Zhejiang2013 are closely related to SARS-CoV-2, and all above viruses are related to or isolated from bats 24. This result suggested that SARS-CoV-2 may be a variant of a bat coronavirus.

In addition, by phylogenetic analysis, we found that Camel_coronavirus_HKU23 should be classified into β-coronavirus, which was classified as genus α-coronavirus in NCBI Nucleotide database before. By literature review, the Camel_coronavirus_HKU23 was recognized as a subgenus of gene recombined β-coronavirus 25. In order to prove the accuracy of the classification of the phylogenetic tree, we added another complete genome sequence of a Camel_coronavirus_HKU23 virus and constructed the phylogenetic tree, and the result supported our conclusion (Figure S1). Therefore, this study corrected the classification errors in NCBI database.

### Variation density and distribution

**Figure 7:**
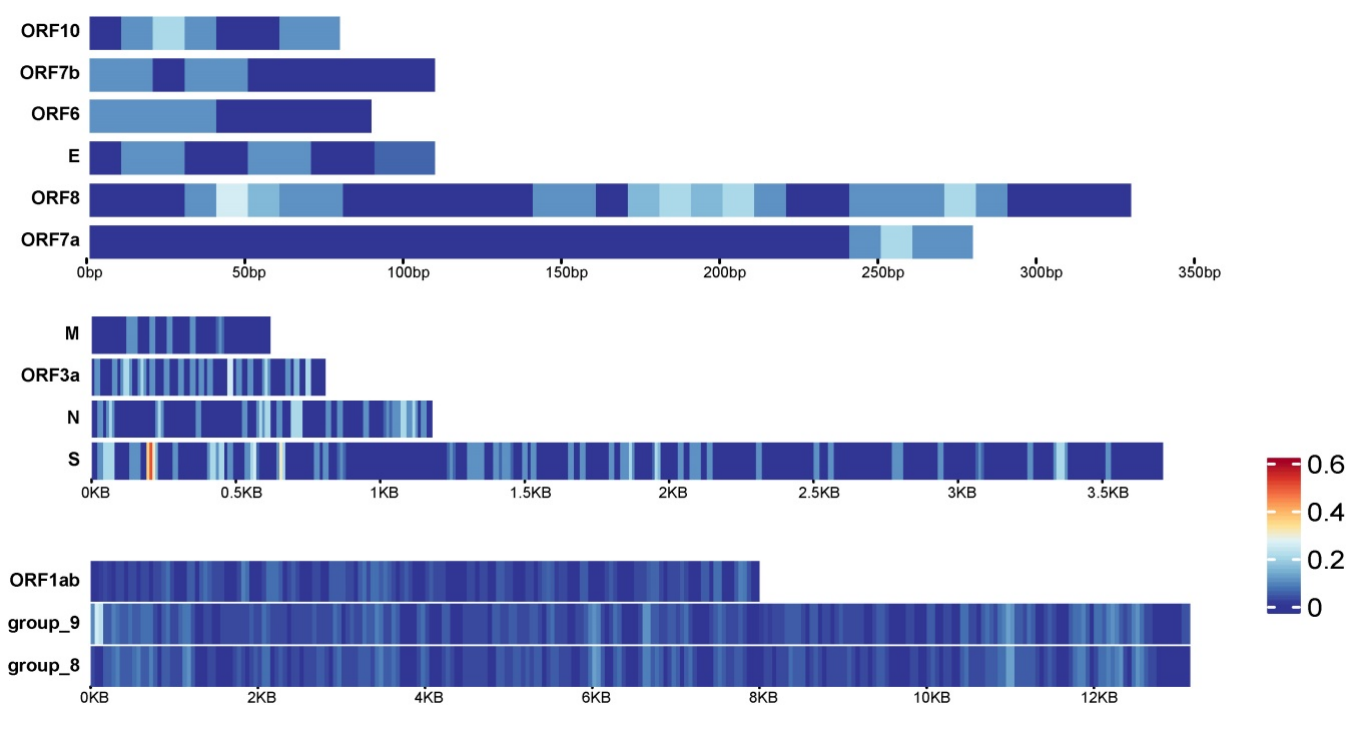
**Variation density and distribution of SARS-CoV-2.** The variation density of 13 genes in SARS-CoV-2 pan-genome. Genes were divided into 3 groups according to their lengths. The group on top are for the shorter genes, and the window size to calculate the variation density is 20 bps. The group in the middle are for medium-length genes, and the window size to calculate the variation density is 20bps. The group listed on bottom are for the longest genes, and the window size to calculate the variation density is 100 bps.

In early 2021, mutations of SARS-CoV-2 have been detected around the world, such as the UK, Europe, the United States and other regions. We collected viruses in early 2020 and in 2021, constructed a pan-genome and used it as the reference genome to compare the virus sequences with the pan-genome using BLAST to detect mutation sites (see Methods for details). There are 13 genes in the pan-genome. We divided them into 3 groups according to their lengths. For long genes, including ORF1ab gene, group_8 gene and group_9 gene, we counted the number of variations in every 100bases as the variation density. For the remaining 11 genes, we detected the variation density using a window size of 20 bases (Figure 7A). It can be found that different genes have different parts that are prone to mutate. For example, the ORF7a gene has no mutations at 0-220bp, but a small amount of mutations occur at 250bp and downstream. The group_9 gene has a higher density of mutations in the starting position. The highest mutation density occurs near the start of S gene.

## Discussion

Since the outbreak of COVID-19 at the end of 2019, genomic research on SARS-CoV-2 has increased significantly. From the published literatures, most of the coronavirus genome research focused on some of the genes, such as ACE2 or a small number of genomes 15-17, 26, 27. There are also studies that compare viral genomes with possible host genomes to infer the virus's host or its intermediate host and predict the origin and spread of the virus 15, 28. Castells M et al. conducted component analysis and Bayesian merger analysis on the complete genome sequences of the SARS-CoV-2 strains recently isolated in Europe, North America, South America and Asia, and found that SARS-CoV-2 has a high evolution rate and rapid characteristics of population growth 29. He and colleagues found that porcine delta coronavirus (PDCoV) may originate from a host switching event between birds and mammals 30. During the transmission between different animals, the adaptive evolution of viruses occurred 30, 31. By genome comparison analysis, Liu and colleagues found three coronaviruses isolated from wild pangolins showed similarity to SARS-CoV-2 and bat coronaviruses, and proposed that pangolin is the natural host of β-coronaviruses 32.

In this study we first introduce the concept of pangenomics into the study of coronavirus family. So far, no pangenomic analysis of coronavirus family has been reported yet. From 3,932 whole genome sequences of coronaviruses in public database, we constructed the pan-genome of coronavirus family, and found a total of 181 genes. Among these 181 genes, only 1 gene (S gene) is core gene (>99%) and 2 genes (M and N genes) are highly conserved genes, which account for 1.66% of the total number of genes. We further calculated the Jaccard similarity between genomes of coronavirus family, which showed significant difference between the intra-species Jaccard similarity and inter-species Jaccard similarity (Wilcoxon test, p<0.0001, Table S3). In addition, the open pan-genome phenomenon implied that with the time progress, as well as the across-species spreading of viruses, some new viral genes will appear. It means the pan-genome will become larger. The appearance of new genes may lead to increased pathogenicity, or decreased pathogenicity. All of the above suggested that the genomes of coronavirus' family have a low conservation level compared with pan-genomes of prokaryotes or eukaryotes.

For SARS-CoV-2 pan-genome, there are 13 core genes. Based on our analysis, S, M, N genes are core genes for SARS-CoV-2, which are also highly conserved for the whole coronavirus family. So, these genes could be noticed as targets in developing nucleic acid diagnostic reagents to decrease the possibility of cross-reaction with other coronavirus species. However, compared to non-coronavirus RNA viruses, which contains no core genes (Table S5), the diversity level of SARS-CoV-2 is much lower. The pan-genome of SARS-CoV-2 containing 13 core genes indicated that the development of vaccines could be easier and the effectiveness of vaccine could be higher than those for other types of RNA viruses.

In order to evaluate the reliability of the pangenomics analysis, we conducted an ortholog analysis and found three highly conserved genes in the coronavirus family. They are the S gene, M gene, and N gene. This ortholog analysis result is highly consistent with the results of our pangenomic analysis. In addition, both methodologies of pangenomic analysis and ortholog analysis showed that the genome of SARS-CoV-2 virus has the highest similarity with the genomes of bat coronaviruses.

Another finding was that we reclassified several coronaviruses in NCBI database. For example, Camel coronavirus HKU23 was previously classified into α-coronavirus genus in NCBI database, and we classified it into β-coronavirus genus. Actually, our finding was supported by So et al^25^. In our recent review, the NCBI database has reclassified it as β genus, proving that our classification is correct. In addition, Swine Acute Diarrhea Syndrome related coronavirus (SADSr-CoV) was uploaded as unclassified virus. We classified it into α-coronavirus genus, which showed the highest similarity to Swine Acute Diarrhea Syndrome coronavirus (SARDS-CoV). Recently, Zhou and co-workers also proposed the sequence similarity of these two virus genomes is as high as 98%^33^. Moreover, another two unclassified viruses Rodent Coronavirus and Bat Coronavirus in NCBI database were reclassified into β-coronavirus genus based on two methodologies.

Based on the current analysis results, the high-incidence region of group_9 gene is the starting position of the gene. Similarly, the starting position of the S gene is most likely to mutate compared to other positions (Figure 7). Therefore, although the occurrence of mutation is random across the genome in general, there are some specific areas we can focus on to detect whether the mutations in these areas have caused the changes in the physical and chemical properties of the virus.

## Materials and Methods

### Data collection

A total of 3,932 complete genomes were downloaded from https://www.ncbi.nlm.nih.gov/nuccore on 24 April 2020. These coronaviruses come from 101 coronaviruses species, including 3 species of unclassified genera. We created a “101 data set” by selecting a genome from each species. We also downloaded the protein sequences of “101 data set” from the Nucleotide database of NCBI.

There are also 23,539 complete genomes for SARS-CoV-2, which were uploaded by different countries and downloaded from https://www.ncbi.nlm.nih.gov/nuccore on 14 July 2021.

In the 23,539 SARS-CoV-2 genomes, we picked at least one sequence for each country with sequences submitted before April 15, 2020. There were 11 genomes in total. In addition, on February 15, 2021, we downloaded 38 more SARS-CoV-2 genome sequences (at least one sequence for each country) collected in 2021 from the same website as above. These 49 SARS-CoV-2 genomes (38 from 2021 and 11 from early 2020) were used for mutation comparison.

Four RNA virus species (Table S5) other than coronavirus were downloaded from https://www.ncbi.nlm.nih.gov/nuccore for comparison of diversity level between SARS-CoV-2and other non-coronavirus RNA viruses.

### Pangenomics analysis of coronaviruses family

We used Roary (version 3.11.2) 34 for pangenomics analysis of coronaviruses. In core gene analysis, considering that the coronaviruses of different genera vary greatly, and the identified core genes are sensitive to the threshold of sequence identity percentage, we set the identity percentage threshold from 25% to 95% with 5% as the step size in Roary. Due to the large variation among different genera, we set the threshold as 30% 35. In order to eliminate the potential annotation errors or genome sequence assembly, genes shared by at least 95% of strains are annotated as “soft core genes”, which was widely adapted in pan-genome analysis 36. Genes shared by more than 99% strains are defined as core genes in some pan-genome analyses 37. We adapted these definitions in this paper. In addition, we defined genes that occur in more than 97% of the genomes as highly conserved genes. In gene presence and absence analysis, the Jaccard similarity between genera was calculated. The Jaccard similarity coefficient J (A, B) of two sets A and B is defined as the intersection size of the two sets divided by the union size of two sets (Eq. 1). The genome sequences are clustered based on the Jaccard similarity, and the genetic relationship of the genera are presented using heat maps.

J (A, B) = ¦A∩B¦/¦A∪B¦=¦A∩B¦/(¦A¦+¦B¦-¦A∩B¦) (1)

We also applied similar pan-genome analysis for RNA viruses such as HIV1, Hepatitis Virus C, Rhinovirus, and Enterovirus C. We counted the core genes and distributed genes number. Compared with SARS-CoV-2, there were no core genes in other RNA virus (Table S5).

### Pangenomics analysis of SARS-CoV-2 coronavirus

A total of 23,539 SARS-CoV-2 sequences were included in this analysis. Since all 23,539 SARS-CoV-2 are from human hosts, there are few sequence differences, we set the threshold of percentage identity to start at 80%. Then, the Roary pipeline was processed with percent identities from 80% to 95%. There is almost no difference between the results using different thresholds, so we set the threshold as 95%.

### The ortholog analysis of coronavirus family

The OrthoFinder (version 2.2.6)^23^ pipeline was used in ortholog analysis of coronaviruses from 101 species. The input file contains the protein sequences in fasta format. The evolutionary tree was drawn using the maximum likelihood method of IQ-TREE 36. The bootstrap value is set to 1000, and the resulted evolutionary tree was visualized with iTOL v4 39.

### Variation density and distribution

We used BLAST 2.11.0+ (with default parameters) 40 for sequence alignment. We used the constructed pan-genome as the reference. Taking each viral genome as an input, we counted the mutation sites of each genome relative to the pan-genome according to the BLAST results.

### Data Availability

All data were from NCBI (https://www.ncbi.nlm.nih.gov/nuccore), and the Accession Numbers of the coronavirus family and SARS-CoV-2 in NCBI were listed in Table S1 and Table S4 respectively. The accession numbers of genomes of other RNA virus were listed in Table S5. The accession numbers of sequences used for mutation comparison were listed in Table S6.

## Supplementary Material

Supplementary table 1. Information of 3,932 genomes of coronavirus family, and the selected 101 genomes of 101 species.

Supplementary table 2. Genes in the pan-genome of coronavirus family.

Supplementary table 3. Jaccard similarity coefficient of genomes between 101 species.

Supplementary table 4. Accession ID of 23,539 SARS-CoV-2 genomes.

Supplementary table 5. Information of other RNA virus compared with SARS-CoV-2.

Supplementary table 6. Information of 49 SARS-CoV-2 genomes isolated in early 2020 and 2021.

## Figures and Tables

**Figure 4:**
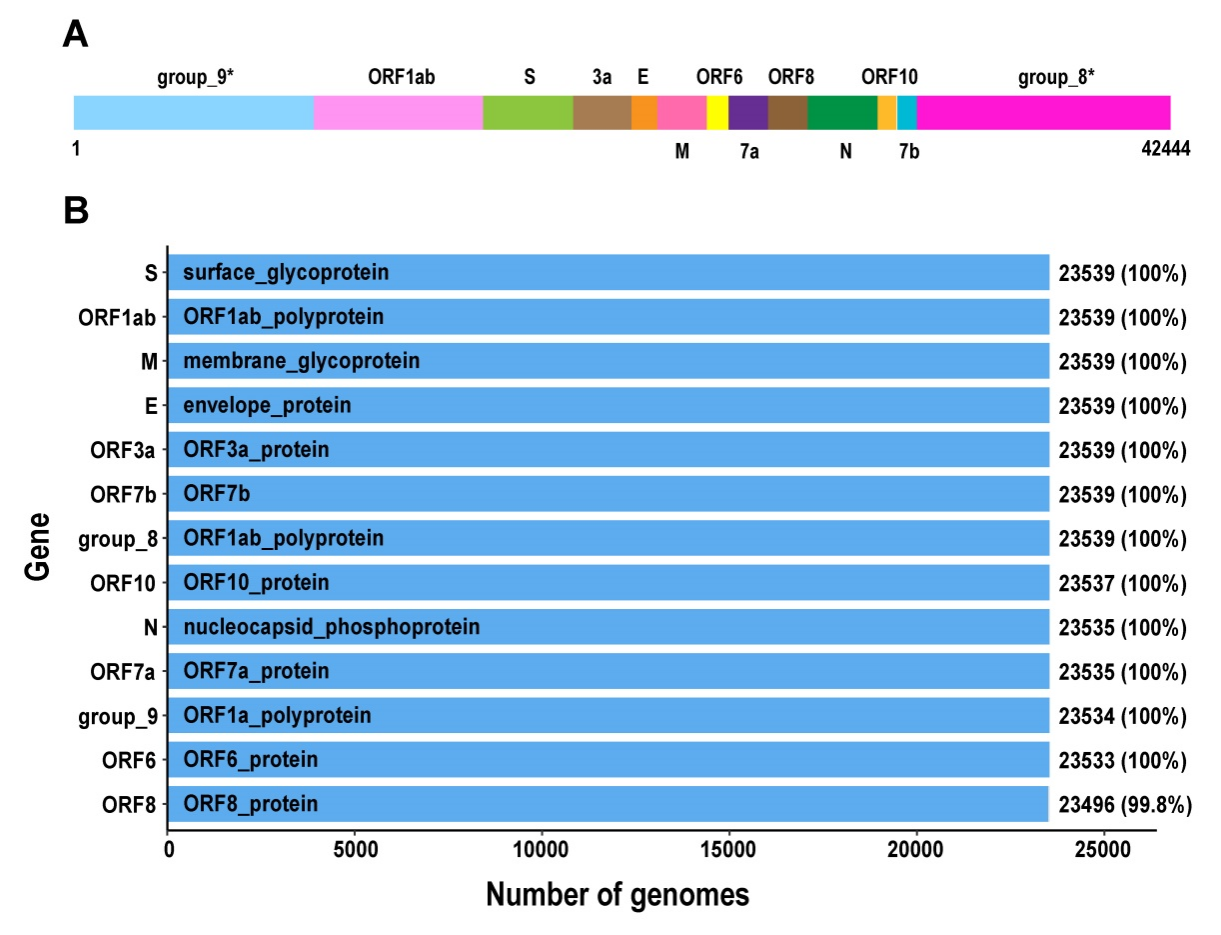
**The pangenomics characterization of SARS-CoV-2**. A. Schematic diagram of the SARS-CoV-2 pan-genome structure constructed under the 95% sequence percentage identity threshold, containing 13 genes and 42,444 bps in length (the names of gene clusters with * are defined by the Roary analysis process). B. All genes in the SARS-CoV-2 pan-genome and the number of samples containing these genes (the total number of samples is 23,539).

**Table 1:** The list of genes present in over 1,000 coronavirus genomes

Gene	Protein	Presence	Genus	Unclassified**
S	spike protein	3,897	αβγδ	Rodent_coronavirusBat_coronavirus SADSr-CoV
M	membrane protein	3,852	αβγδ	
N	nucleocapsid phosphoprotein	3,843	αβγδ	
ORF1ab	ORF1ab polyprotein	3,002	αβγδ	
E	envelope protein	2,267	αβ	Bat_coronavirus
				Rodent_coronavirus
orf1ab	ORF1ab polyprotein	2,333	αβγ	Bat_coronavirus
				SADSr-CoV
ORF3a	ORF3a protein	1,398	β	Bat_coronavirus
ORF6	ORF6 protein	1,397	β	
ORF7a	ORF7a protein	1,394	αβ	
ORF8	ORF8 protein	1,374	β	
ORF10	ORF10 protein	1,163	β	
ORF7b	ORF7b protein	1,318	β	Bat_coronavirus
group_27*	envelope protein	1,032	α	Rodent_coronavirusBat_coronavirus

*group_27 is a gene cluster annotated by Roary pipeline. The encoded protein by group_27 gene cluster is E protein, but their sequence identity is less than 30%. Therefore, it is listed separately as group_27. **The unclassified category contains 3 types of viruses, namely Rodent_coronavirus, Bat_coronavirus, and Swine_acute_diarrhea_syndrome_related_coronavirus (SADSr-CoV).
